# Paediatric Tuberculosis at a Referral Hospital in Istanbul: Analysis of 250 Cases

**DOI:** 10.1155/2016/6896279

**Published:** 2016-05-19

**Authors:** Ozden Turel, Selcen Kazanci, Ismail Gonen, Cigdem Aydogmus, Emel Karaoglan, Rengin Siraneci

**Affiliations:** ^1^Department of Pediatrics, Faculty of Medicine, Bezmialem Vakıf University, Istanbul, Turkey; ^2^Department of Pediatrics, Bakirkoy Sadi Konuk Educational and Research Hospital, Istanbul, Turkey; ^3^Department of Pediatrics, Private Liv Hospital, Istanbul, Turkey; ^4^Department of Pediatrics, Kanuni Sultan Suleyman Educational and Research Hospital, Istanbul, Turkey

## Abstract

*Background*. Tuberculosis (TB) still remains a growing public health problem globally. TB in children is often diagnosed clinically.* Methods*. We conducted a retrospective chart review of children with TB from November 2004 through December 2010 to determine the appropriateness of using contact history and diagnostic testing.* Results*. A total of 250 children with TB were identified. One hundred and sixty-two children had only pulmonary disease while 39 had features of both extrapulmonary and pulmonary TB. Mean age was 7.8 years. Thirty-six patients had known contacts. The index case/cases were first-degree relatives in 75%. Sixteen patients who were symptomless were yielded by contact investigation of newly identified TB cases. Tuberculin skin test positivity was 53.3%. Acid-fast bacilli smear positivity was 13.1%, and culture positivity was 18.7%. Twenty-six patients had histopathology of nonrespiratory specimens (lymph nodes and other tissues) showing granulomatous inflammation and caseous necrosis consistent with TB.* Conclusions*. Presence of contact history directed us to search for TB in children with nonspecific symptoms even if physical examinations were normal. Some children who were close contacts to TB cases were identified to have TB before development of symptoms.

## 1. Introduction

Tuberculosis (TB) still remains a growing public health problem globally. It is the 8th leading cause of death worldwide with 8.7 million new cases and 1.4 million deaths each year [1]. The 2015 Millennium Development Goal of the World Health Organization (WHO) European Region aims to halve the prevalence and number of deaths associated with TB [2]. Turkey is a country with an intermediate prevalence of TB and an incidence rate of 21 per 100,000 inhabitants (44.1 per 100,000 inhabitants in Istanbul) [3].

Childhood TB reflects uncontrolled adult TB in a community [4]. In high disease burden settings, children younger than 13 years of age constitute 13.7% of all cases [5]. An estimated 490,000 TB cases detected among children constitute 6% of all cases in Turkey [2]. Although pediatric TB remains a public health emergency, research and surveillance data have been limited. Pediatric TB poses diagnostic challenges. The microbiological confirmation rate is low, and a diagnosis is generally made using a combination of history, tuberculin skin test (TST), and the chest radiograph data [6]. Here, we determine the appropriateness of using contact history and diagnostic testing in children with TB.

## 2. Materials and Methods

### 2.1. Patient Selection

We reviewed the medical records of children with TB treated at a sentinel hospital in Istanbul from November 2004 through December 2010. Bakirkoy Maternity and Children's Training and Educational Hospital (BEH), a tertiary care hospital with 400 beds, is a reference center for sick children in Istanbul. In the absence of laboratory confirmation, the Center for Disease Control and Prevention (CDC) defines a clinical case as corresponding to a positive TST, signs or symptoms of TB, treatment with two or more anti-TB drugs, and a complete diagnostic evaluation. If all of these criteria are not met, a diagnosis of TB is fulfilled if the patient is being treated with two or more anti-TB drugs determined to be medically justified by the TB Control Surveillance Unit [6]. Among 1109 patient records, 250 children fulfilled these criteria and were included in the study.

### 2.2. Contact History

A household contact is defined as a person who has shared the same enclosed living space as the index case for one or more nights or for frequent or extended daytime periods during the three months prior to the start of the current treatment episode [7]. Contact investigation is the yield of secondary cases among contacts of newly identified TB cases. A source case investigation is conducted to identify an infectious person who might be the source case of someone with TB or a latent infection with* M. tuberculosis*.

### 2.3. Classification Based on Anatomical Site of the Disease

Pulmonary TB (PTB) refers to any bacteriologically confirmed or clinically diagnosed case of TB involving the lung parenchyma or the tracheobronchial tree. Miliary TB is classified as PTB because there are lesions in the lungs. Tuberculous intrathoracic lymphadenopathy (mediastinal and/or hilar) or tuberculous pleural effusion, without radiographic abnormalities in the lungs, constitutes a case of extrapulmonary TB. A patient with both pulmonary and extrapulmonary TB is classified as a case of PTB [7].

### 2.4. Laboratory Investigations

We performed TSTs by placing purified protein derivative (PPD) and 0.1 mL of 5 Todd units intradermally during the initial visit; we then interpreted the results within 48–72 hours. An induration of ≥15 mm in children previously vaccinated with* Bacillus* Calmette-Guerin (BCG) or an induration of ≥10 mm in children not previously vaccinated with BCG was interpreted as positive according to the recommendations of the Turkish Ministry of Health [8]. Microbiological tests included direct microscopy by Ziehl-Nielsen stain for the presence of acid-fast bacilli (AFB),* M. tuberculosis* culture, and nucleic acid amplification tests (NAAT) of clinical samples including sputum, gastric aspirate, cerebrospinal fluid, pleural fluid, and urine. We also recorded the histopathological findings of the tissue specimens.

### 2.5. Statistical Analysis

We used SPSS version 13.0 (SPSS Inc., Chicago, IL, USA) for statistical analyses. All statistical hypothesis tests were two-sided, and a *P* value less than 0.05 was considered to be statistically significant. We used independent samples *t*-tests or a nonparametric analog, Mann-Whitney *U* test, to compare the means of two independent groups. We compared categorical variables using a chi-squared test or Fisher's exact test.

The local ethics committee approved the study, and written, informed consent was obtained from all of the subjects' parents or legal guardians.

## 3. Results

### 3.1. General Demographic Data

Among 250 children with TB (age range: 2–179 months), 62.9% were hospitalized. The cohort was roughly equal in gender proportions (126 females, 124 males). Pulmonary TB was the most common diagnosis (80%). Among them 162 patients had only pulmonary disease and 39 patients had both pulmonary and extrapulmonary involvement. Eight patients had severely disseminated diseases, including TB meningitis and miliary TB (Table 1).

### 3.2. Contact History and Vaccination Status

Based on parental reports, 157 (63.2%) patients had a family history of TB. An infectious person who might be the source case was identified for 36 patients: 12 fathers, 9 mothers, 4 siblings, 4 aunts/uncles and 2 grandfathers/grandmothers, and 5 multiple contacts (Table 2). Drug-susceptibility results were not available for all of the contacts, but 10 of the contacts were reported to have drug resistance to at least one drug: 2 only to isoniazid (INH) and 1 to INH and streptomycin (SM); 6 were reported to be multidrug resistant (MDR), and 1 was reported to be resistant to 4 drugs (INH, rifampicin, pyrazinamide, and SM). Thirty-one patients (12.7%) had previously received INH prophylaxis either for a latent TB infection or for a household contact with an adult with active TB. Few of these were relapse cases and 9 (29%) had recent contact to an infectious TB case (4 of the contacts were drug resistant TB).

Nearly three-quarters of patients (70.2%) had BCG scars (65.9% had 1 scar and 4.1% had at least 2 scars). Sixty-one patients did not have any scars even though they had been administered a BCG vaccine.

### 3.3. Clinical Features of the Patients

Prolonged cough persisting for more than 2 weeks (69.5%) and fever (43.1%) were the most common symptoms (Table 3). Thirty-eight (15.1%) patients presented with recurrent pneumonia. Twenty patients had no symptoms and had normal physical examination findings. Among these 20 patients, only 4 had an unknown or a negative contact history: two patients had nephrotic syndrome and TST positivity. Their chest X-rays revealed pathological findings and anti-TB treatment was initiated. One patient was evaluated for calcified cysts in the liver and spleen. His contact history and TST status were unknown, but his chest X-ray was pathologic. A fourth patient had a negative contact history and TST, but his chest X-ray revealed lobar consolidation. All of the remaining 16 patients were diagnosed following contact investigation. Nearly half of the patients (56%) were at or under 5 years of age and had a positive TST (80%). Radiological examination revealed intrathoracic lymphadenopathy (LAP) (2 patients), LAP+ consolidation (9 patients), nodular infiltrations (4 patients), and cavity formation (1 patient).

### 3.4. Diagnostic Tools Used with the Patients

A TST was performed in 210 (84.4%) patients and was positive in 112 (53.3%) cases. Microbiological evaluation (direct microscopy and/or culture) was available for 122 (48.8%) patients; AFB staining positivity was found in 13.1% patients and culture positivity was recorded in 18.7% of patients. Thirty-three patients had NAAT results, and 48.5% of these results were positive. Gastric aspirate was the most commonly studied specimen for identifying TB bacilli. One patient had a positive growth in his sputum sample, and another had a positive growth in his mediastinal LAM biopsy. No significant difference in microbiological confirmation rate was detected between patients with PTB and those with extrapulmonary TB (Table 4). Twenty-six patients had histopathology of nonrespiratory specimens (lymph nodes and other tissues) showing granulomatous inflammation and caseous necrosis consistent with TB.

### 3.5. Radiological Findings

**Figure 1:**
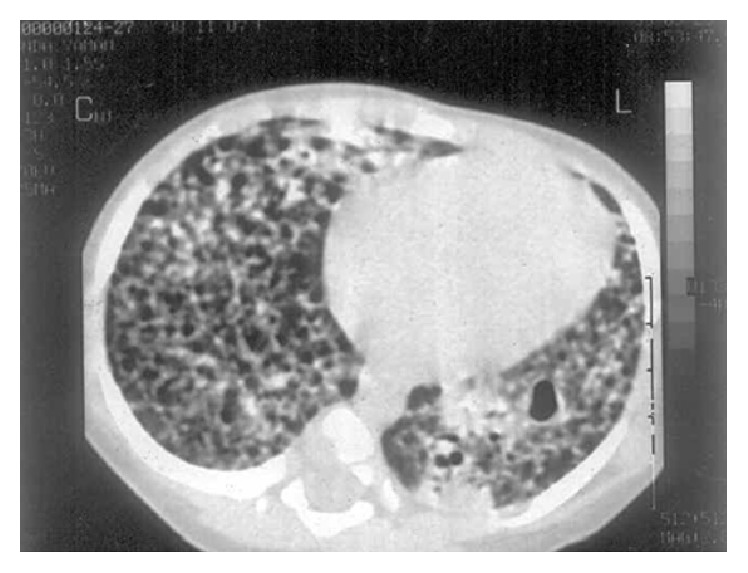
Miliary pattern with cavitary formation.

**Figure 2:**
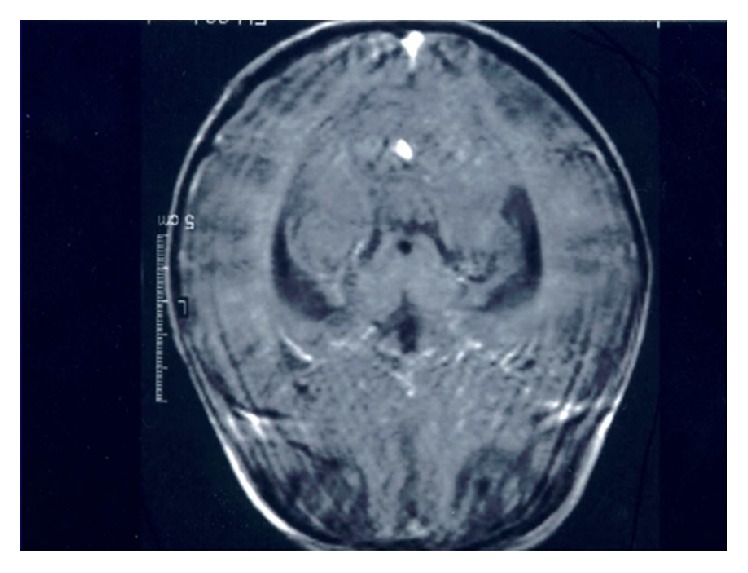
Basilar enhancement on cranial MRI.

**Figure 3:**
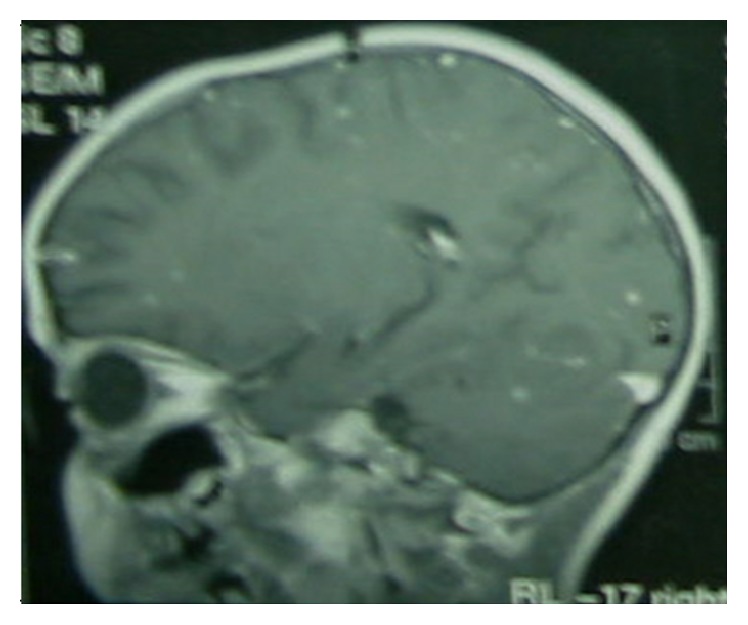
Tuberculomas on cranial MRI.

Radiological findings of the chest revealed 6 independent events (Table 5). Intrathoracic (hilar or mediastinal) LAP and parenchymal infiltrates were the most common findings (46%). Eighteen (7.2%) patients had only intrathoracic (hilar or mediastinal) LAP. Eight patients had extensive pulmonary involvement (Figure 1). All of the patients with TB meningitis had abnormal findings on cranial magnetic resonance imaging, including basilar enhancement and tuberculomas (Figures 2 and 3).

## 4. Discussion

Childhood TB has some unique features; it is less often bacteriologically confirmed compared with adult TB cases due to its paucibacillary nature, and it is associated with a greater likelihood of extrapulmonary and disseminated presentations. In childhood TB cases, it is also often difficult to obtain appropriate clinical specimens [5, 6]. Extrapulmonary tissues were involved in 35% of cases in the current study, which was a higher rate than that reported by a Taiwanese study (24.8%) and lower than reports from Ethiopia (47.4%, 40%) and India (46%) [9–11]. The risk of progression to disease following infection is greatest among the very young. Nearly one-fifth of children in our cohort with extrapulmonary TB were under 5 years of age. A previous study evaluating 2205 children with TB also showed that 62% of patients were under 6 years old [12]. Mtabho et al. evaluated 1615 children with TB in Kilimanjaro and showed that 49.3% of patients were under 5 years old [13].

Diagnosing TB in a child is often based on the presence of the classic triad: (1) recent close contact with an infectious case, (2) a positive TST or interferon-gamma release assay, and (3) suggestive findings on a chest radiograph or upon physical examination [14]. In setting with a high prevalence of TB and human immunodeficiency virus (HIV), clinical predictors of culture-confirmed pulmonary TB were found to be prolonged fever and chest X-rays suggestive of TB or a positive TST [15]. Contact history was present in 62% of cases but was not significantly associated with definite TB. In countries with a low endemicity of TB, contact history is more important. A 20-year study conducted in Italy showed that a history of contact with a patient with active TB was significantly associated with pulmonary TB [16]. Young children living in close contact with a source case of smear-positive pulmonary TB are at particular risk for TB infection and disease. World Health Organization guidelines advise that all children aged 0–4 years, regardless of symptoms, and children aged 5 years and above who are symptomatic and have been in close contact with a TB case be evaluated for TB [7]. A household contact is often found to be the source of infection in children under 5 years of age with TB; infants and young children are particularly likely to have contracted TB at home. Wu et al. reported that among 1212 pediatric patients with TB, 364 (31%) patients had a contact history at home [17]. These authors suggest that children with a contact history at home have a higher chance of contracting severe TB. Ilgazli et al. reported that among 636 cases with extrapulmonary TB diagnosed between 1996 and 2000 in Kocaeli, Turkey, a contact history was determined in 242 cases (38%); of these, 194 individuals were younger than 15 years of age (80.2%) [18]. Tanir et al. evaluated 118 children with TB in Ankara, Turkey, between 2001 and 2003. A history of contact with an adult having TB was observed in 58% of cases [19]. An index case was determined in 214 out of 539 children with TB (39%) in a multicenter study [20]. In this study, we asked the parents whether there was a person with TB in their family and 63.2% answered “yes.” However, not all of these cases were newly diagnosed cases. An index case(s) could be detected in 36 patients. The incidence of TB in Istanbul is higher than Turkey's average. The annual number of contacts examined in TB dispensaries per TB patient has been reported to be 5.5, and the total number of persons receiving prophylaxis has been reported to be 5,283 [21].

Our study revealed 53.3% positivity of TSTs, similar to that of Pekcan et al. (55.3%) [20]. Tuberculin skin test positivity may be even lower in malnourished children [10]. Tuberculin skin test results may vary significantly depending on the time of measurement, the type of PPD used, and the method of measurement. There are a number of methods for performing TSTs, but the Mantoux method is recommended. The TST should be standardized for each country using either 5 tuberculin units (TU) of tuberculin purified protein derivative (PPD-S) or 2 TU of tuberculin PPD RT23, which yield similar reactions in children infected with* M. tuberculosis*. A combination of cough, fever, and night sweats has been reported to be the most common clinical finding in TB [6]. In a study involving 13 infants with PTB, night sweats and fever were reported in 35.7% and 21.4% of patients, respectively. Four out of the 13 patients (30%) were asymptomatic, and physical examinations were normal in 12 patients (92%) [22]. In our study, the most common symptoms were a cough (69.5%), a long-standing fever (43.1%), and night sweats (26.2%). Up to 40% of patients had normal findings upon physical examination. Sixteen patients who were symptomless were yielded by contact investigation of newly identified TB cases.

Despite minimal clinical findings, radiographic imaging may reveal the extent of TB. In Iran, pulmonary radiologic features were studied in 70 children confirmed to have TB over a five-year period [23]. The most common feature was hilar/mediastinal LAP (85%). Children younger than 3 years of age had the highest rate of lymph node involvement, but parenchymal lesions were less frequent compared in these young individuals than in older children. In this study, 18 patients (7.2%) had solely intrathoracic LAP.

Gastric aspiration remains the most common method for obtaining respiratory samples from children. However, cultures of gastric aspirate specimens are positive for TB in only 30–40% of cases [24]. Smears are even less reliable, with positive results in fewer than 10% of cases. Only 13.6% of the patients were microbiologically confirmed as having TB in this study. Hailu et al. detected smear positivity in 9.6% of pediatric TB cases [25]. Studies from Turkey have revealed smear positivity ranging from 23.5 to 31% in children with TB [26, 27]. Although bacteriological confirmation of TB is not always possible, every effort should be made to confirm a diagnosis of TB in a child. Different fractions of tuberculosis laboratories offer different diagnostic tests in Turkey: microscopy (416 laboratories; 99.8%), conventional culture (165 laboratories; 39.6%), liquid culture (79 laboratories; 18.9%), species identification (45 laboratories; 15.3%), and drug-susceptibility testing (69 laboratories; 16.5%) [21]. Liquid culture systems and molecular line probe assays for rapid detection of MDR TB have been endorsed by the WHO [7]. World Health Organization-endorsed genotypic (molecular) testing (i.e., Xpert MTB/RIF) is useful for the rapid diagnosis of TB and the identification of rifampicin resistance. Xpert MTB/RIF assays are recommended as an initial test for diagnosing tuberculous meningitis.

## 5. Conclusions

Almost two-thirds of children with TB had a family history of TB and 36 patients had known contact with a person with contagious TB. Presence of contact history directed us to search for TB while evaluating children with nonspecific symptoms even if physical examinations were normal. Additionally some children who were close contacts to adult TB cases were identified to have TB before development of symptoms. We emphasize that a history of recent close contact with an infectious case of TB, especially a first-degree relative, is of paramount importance when diagnosing TB in children. When managing patients with a cough and fever, the presence of intrathoracic LAP in chest X-rays should alert physicians about TB, even if no infiltration is detected in the lung parenchyma. The confirmation rate via smear and culture is low, particularly for extrapulmonary TB. Molecular tests may provide evidence for early treatment.

## Figures and Tables

**Table 1:** Clinical presentation of children with TB.

TB disease sites	Number (%)
*Pulmonary*	*162 (64.8)*
*Extrapulmonary*	*49 (19.6)*
Peripheral lymphadenitis	18 (36.7)
Pleural effusion	11 (22.4)
Intrathoracic lymph node	10 (20.4)
Peritoneum	3 (6.1)
Meningitis	3 (6.1)
Kidney	2 (4.9)
Pericarditis	1 (2)
Uveitis	1 (2)
*Pulmonary + extrapulmonary*	*39 (15.6)*

**Table 2:** Index cases identified.

Index case	Number
Father	12
Mother	9
Sibling	4
Uncle/aunt	4
Grandfather	1
Grandmother	1

Multiple contacts^*∗*^	5

^*∗*^Both parents 1; sibling and grandfather 2, uncle and father and grandfather 1, and father and aunt 1.

**Table 3:** Clinical features of children with TB.

	Number (%)
*Symptoms*	
Cough	173 (69.5)
Fever	107 (43.1)
Night sweating	65 (26.2)
Weight loss	61 (24.8)
Wheezing	31 (12.5)
Sputum production	17 (6.9)
Hemoptysis	7 (2.8)
Convulsions	1 (0.8)
*Signs*	
Pulmonary rales	77 (32)
Decreased or absent breath sounds	56 (22.9)
Focal neurologic signs	1 (0.8)

**Table 4:** Positive diagnostic tests in patients with TB.

Test	Pulmonary	Extrapulmonary	*P* value
	*N* (%)	*N* (%)	
TST	87/170 (51.2)	25/40 (62.5)	0.97
MTB culture	17/80 (21.2)	3/27 (11.1)	0.24
AFB stain	14/91 (15.4)	2/31 (6.5)	0.20
PCR	13/24 (54.2)	3/9 (33.3)	0.28
Histopathology	13/15 (86.7)	13/13 (100)	0.17

AFB: acid-fast bacillus and PCR: polymerase chain reaction.

**Table 5:** Main radiographic findings in pulmonary imaging.

Pathology	*N* (%)
Mediastinal or hilar LAP	122 (50)
Lobar consolidation	121 (49)
Patchy consolidation or nodular opacities	53 (21)
Pleural effusion	32 (13)
Cavitary formation	15 (6)
Miliary tuberculosis	8 (3)
